# Parentage-Based Group Composition and Dispersal Pattern Studies of the Yangtze Finless Porpoise Population in Poyang Lake

**DOI:** 10.3390/ijms17081268

**Published:** 2016-08-11

**Authors:** Minmin Chen, Yang Zheng, Yujiang Hao, Zhigang Mei, Kexiong Wang, Qingzhong Zhao, Jinsong Zheng, Ding Wang

**Affiliations:** 1 The Key Laboratory of Aquatic Biodiversity and Conservation of the Chinese Academy of Sciences, Institute of Hydrobiology of the Chinese Academy of Sciences, Wuhan 430072, China; chenminminok@163.com (M.C.); zhengyang8235@163.com (Y.Z.); hao.yj@ihb.ac.cn (Y.H.); pandameizhigang@gmail.com (Z.M.); wangk@ihb.ac.cn (K.W.); zhaoqz0517@163.com (Q.Z.); 2 Research Center of Aquatic Organism Conservation and Water Ecosystem Restoration in Anhui Province, School of Life Sciences, Anqing Normal University, Anqing 246133, China; 3 University of the Chinese Academy of Sciences, Beijing 100049, China

**Keywords:** *Neophocaena asiaeorientalis asiaeorientalis*, parentage identification, social structure, matrilineal, dispersal pattern

## Abstract

Social behaviors are poorly known for the critically endangered Yangtze finless porpoise (YFP, *Neophocaena asiaeorientalis asiaeorientalis*). Here, group composition and dispersal patterns of the YFP population living in the Poyang Lake were studied by parentage-based pedigree analyses using 21 microsatellite loci and a 597 bp segment of the mitochondrial DNA control region. In this study, 21 potential mother-offspring pairs and six potential father-offspring pairs (including two potential parents-offspring pairs) were determined, among which 12 natural mother-offspring groups and a maternal group of three generations were found. No genetically-determined fathers were found associated with their offspring. This study also found that maternally related porpoises at the reproductive state tend to group together. This suggest maternal relationship and reproductive state may be factors for grouping in the YFP population. In natural mother-offspring groups, male offspring were all younger than two years old, which suggest male offspring may leave their mothers at approximately two years of age, or at least they were not in tight association with their mothers as they may have been under two years old. However, female offspring can stay longer with their mothers and can reproduce in the natal group.

## 1. Introduction

The social behavior of cetaceans is both complex and interesting. Advances in molecular techniques have led to an increasing number of studies that combine molecular, observational, and photo-ID data to reveal a variety of grouping and dispersing patterns in cetacean species [1,2]. Various cetacean social behaviors have been reported, including fluid fission-fusion societies described in small delphinid species (e.g., bottlenose dolphins (*Tursiops aduncus*) [3]; spinner dolphins (*Stenella longirostris*) [4]), matrilineal groups in larger toothed whales (e.g., killer whales (*Orcinus orca*) [5]; sperm whales (*Physeter macrocephalus*) [6]), and associations among individuals of both sexes or just a single sex that vary in size (from few to hundreds of individuals), duration (temporary or permanent), and composition (single or multiple generations) of Atlantic white-sided dolphins (*Lagenorhynchus acutus*) [7]. The social behavior of freshwater dolphins was also observed. Smith and Reeves [8] reviewed that Amazon River dolphins (*Inia geoffrensis*) sometimes form loose fishing groups and male-on-male aggression is common. Irrawaddy dolphins (*Orcaella brevirostris*) form fission-fusion group dynamics with frequent social interactions and cooperative feeding, and mother-young associations can be observed in the Ganges and Indus dolphins (*Platanista* spp.). Still, compared to marine cetaceans, knowledge about the social behavior of freshwater cetaceans remains very limited.

The Yangtze finless porpoise (YFP, *Neophocaena asiaeorientalis asiaeorientalis*) is a small, freshwater toothed whale that occurs only in the middle and lower reaches of the Yangtze River (from Yichang to Shanghai) and its adjoining lakes (Poyang and Dongting) [9]. Due to its small population size, sharply declining population, and high probability of extinction, the YFP was recently reclassified a Critically Endangered (CR) population in the International Union for the Conservation of Nature (IUCN) Red List of Threatened Species [10,11]. Since the YFP is notoriously difficult to identify and track using normal observation methods (e.g., visual surveys and photo-identification), primarily due to their small size (≈1.5 m in length), lack of a dorsal fin, and behavior (e.g., only surface for 1–2 s at a time), little is known about their group composition and dispersal patterns. Understanding the social behaviors of the YFP will be instrumental for effective conservation and contribute to our general knowledge of the social behaviors of freshwater cetaceans [12,13,14].

Previous research suggests the YFP in the Yangtze main stream live either alone, or in groups of 2–20 individuals [15,16]. Groups consisting of two to three individuals have been suggested to be the most common and are generally called “core units”. Groups of >20 individuals are rare and likely consist of several core units. Similar grouping patterns have also been reported for porpoises living in semi-natural reserves [17,18,19]. Due to the limits of observational data, none of these previous studies have analyzed genetic relationships among individuals within YFP groups, nor investigated dispersal patterns.

Poyang Lake is the most important limnic habitat of the YFPs with a population of ≈450 individuals (almost 50% of the total population of the YFPs [20]). To investigate the health and social structure of the Poyang Lake population, four capture-release surveys were conducted in the spring of 2009, 2010, 2011, and 2015, during which all captured individuals were marked with an internal ID and genetic samples were obtained. This dataset offers a unique opportunity to study aspects of social behaviors using both genetic and observational data. In this study, we used observational data collected during these four capture surveys and genetic data obtained from 21 microsatellite loci and a 597 bp highly variable segment of the mitochondrial DNA control region, to study the relationships of individuals in natural groups and the dispersal patterns of the YFP population living in the Poyang Lake.

## 2. Results

### 2.1. Genetic Variation

A total of 171 alleles were detected at 21 microsatellite loci among 122 individuals. No evidence was found for null alleles, stuttering and allele dropout in each locus by the program MICRO-CHECKER, at a confidence level of 95%. The number of alleles (*N*_a_) ranged from 4 to 16 (mean 8.1), with observed heterozygosity (*H*_o_) ranging from 0.374 to 0.854 (mean 0.661) and expected heterozygosity (*H*_e_) ranging from 0.361 to 0.828 (mean 0.674; Table 1). The polymorphic information content (*PIC*) ranged from 0.345 to 0.801 (mean 0.629; Table 1). The combined non-exclusion probability for one candidate parent (*N*_e-1p_) and for one candidate parent given the genotype of a known parent (*N*_e-2p_) were 7.20 × 10^−4^ and 2.17 × 10^−6^, respectively (Table 1). In other words, the total exclusion probability of the combined loci when no parents were known (PE II) was 99.93%, and when one parent was known (PE I) was 99.99%. No deviation from Hardy-Weinberg equilibrium was detected for each locus. The inbreeding coefficient index (*F*_is_) of each locus ranged from −0.182 to 0.178, with an average of 0.0002 (*p* > 0.05).

Among the 122 individuals, three mitochondrial DNA (mtDNA) haplotypes were found: NAACR-Hap1, NAACR-Hap2, and NAACR-Hap8 (GenBank accession number: KC135874, KC135875 and KC135881). NAACR-Hap1, NAACR-Hap2 and NAACR-Hap8 were shared by 53, 64, and five individuals, respectively (Table S1).

### 2.2. Parentage Assignment and Relatedness

**Figure 1:**
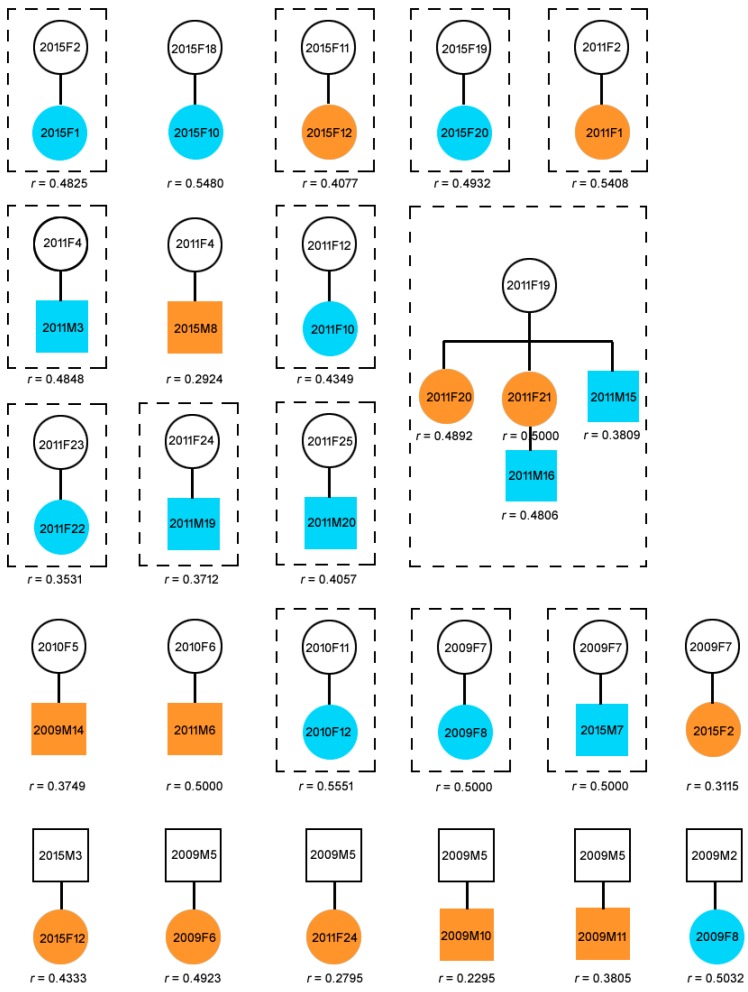
Twenty-one mother-offspring pairs and six father-offspring pairs detected by CERVUS in the Yangtze finless porpoise population living in Poyang Lake. Parent-offspring pairs in dotted boxes were natural groups. The maternal line consisting of three generations was a natural maternal group captured in a sandpit in 2011. Blue represents offspring younger than two years old. Orange stands for offspring older than two years old. Pairwise relatedness index *r* was calculated by the triadic likelihood estimator (TrioML).

Twenty-one potential mother-offspring pairs and six potential father-offspring pairs (including two potential mother-father-offspring families: 2015F11-2015M3-2015F12, 2009F7-2009M2-2009F8) were detected by CERVUS (Figure 1). 

Among the detected mother-offspring pairs, ten female offspring ranging 0.1–9.5 years old, were found associated with the mother according to the field observation record, of which four were aged >2 years old (Figure 1, Table S2), while six male calves ranging 0.3–1.7 years old were found associated with the mother (Table S2). Two female individuals, 2015F2 (5.2 years old) and 2015F10 (1.8 years old), and three male individuals, 2009M14 (6.8 years old), 2015M8 (5.4 years old), and 2011M6 (3.7 years old), were found not associated with the mothers (Table S2). All mothers and their offspring shared the same mtDNA haplotype (Table S2). No genetically-determined fathers were found associated with their offspring (Table S2). The mean pairwise relatedness index (*r*) value for the potential mother-offspring pairs was 0.4479 (0.2924–0.5551), and for the potential father-offspring pairs was 0.3863 (0.2295–0.5032) (Figure 1). The mean overall *r* value among all adults and among all individuals was 0.039 and 0.040, respectively. In general, individuals with relatedness values larger or equal than half-siblings (half-sibs) (the theoretical *r* ≥ 0.25) are deemed related, while all other individuals remain unrelated (the theoretical *r* < 0.25) [21]. However, in natural populations, the *r* values estimated from microsatellite loci may fluctuate around the theoretical value. Blouin et al. [22] suggested using the midpoint between the means of the two distributions as the cut-off value for classification. For example, if one wanted to distinguish between half-sibs (the theoretical *r* = 0.25) and lower grade related individuals (the theoretical *r* = 0.125) in a natural population, the cut-off value should be 0.1875. That means individual pairs with *r* ≥ 0.1875 would be classified as belonging to the category whose relatedness is larger than half-sibs. Using the cut-off values of *r* ≥ 0.1875, we found that 4.48% of all pairs of individuals were related to some extent.

### 2.3. Composition of Natural Groups

During all four capture-release surveys (particularly those conducted in 2009 and 2010 in the channel), human disturbance due to the use of chasing boats and noise may have separated or centralized porpoise individuals. Therefore, most large groups captured in a single net were unlikely to be natural groups. In 2011 and 2015, the survey was conducted in sandpit areas. No chasing boats were used. Hence, human disturbance and sampling bias were largely reduced. We were fortunate enough to detect a maternal line consisting of three generations in a single group captured in 2011 (Figure 1). There are four mother-offspring pairs detected in this maternal line, and all members in this maternal line shared the same mtDNA haplotype (NAACR-Hap2; Table S1). Based on the detected genetic relationship and the observation data which showed that they grouped together (Table S2), we treated members in the maternal line as a natural group. Additionally, twelve mother-offspring groups (groups 2015F2-2015F1, 2015F11-2015F12, 2015F19-2015F20, 2011F2-2011F1, 2011F4-2011M3, 2011F12-2011F10, 2011F23-2011F22, 2011F24-2011M19, 2011F25-2011M20, 2010F11-2010F12, and 2009F7-2009F8, Group 2009F7-2015M7) were further detected (Figure 1). These were all considered to be natural mother-offspring groups based on a high degree of behavioral interaction (field observation data), and genetic relatedness.

## 3. Discussion

### 3.1. Parentage and Relatedness

When using microsatellite markers for paternity analysis, the credibility of the results is highly dependent on the exclusion probability [14,23]. As revealed by previous studies, paternity results are credible when PE I and PE II values exceeded 99.9% and 99%, respectively [14,23,24,25,26]. In this study, PE I and PE II values were 99.99% and 99.93%, respectively, indicating that the 21 microsatellite loci we used were appropriate for parentage analysis. This was also supported by the fact that nearly all suspected mother-offspring pairs (those observed together and captured in one net) were genetically assigned mother-offspring status (Figure 1, Table S3).

In recent years, serious human interference within the estuary area of the Poyang Lake (e.g., the construction of two bridges across the outlet area, and numerous sand-transport vessels) has caused the river-lake migration of the YFPs to be largely restricted. Thus, inbreeding within the Poyang Lake population has become a matter of great concern. The genetic diversity of this population, with a mean *H*_o_ = 0.661 and a mean *H*_e_ = 0.674, is moderate compared to other cetaceans [27,28]. Our relatedness analysis revealed that there is no genetic signature of inbreeding in the YFP population living in Poyang Lake. First, no significant *F*_is_ was detected in this study. Second, our relatedness index did not reflect inbreeding. In a free-range population, the average *r* between potential parents should be close to zero (e.g., −0.014 in *Ursus arctos*, [14]; 0.056 in *Ursus americanus*, [13]), and we found that the mean *r* between potential parents was very low (0.039). Third, as reported by Csilléry et al. [21], for natural populations, we found that the ratio of individual pairs with some relatedness was <10% (4.4%), further indicating little inbreeding in this population currently. Conversely, the population in Tian’ezhou ex situ reserve demonstrates 26.14% of individual pairs have some relatedness (*r* > 0.1875) [29]. Chen et al. [29] inferred this ex situ population was probably in high risk of, or has already been suffering from, inbreeding.

### 3.2. Group Composition

**Figure 2:**
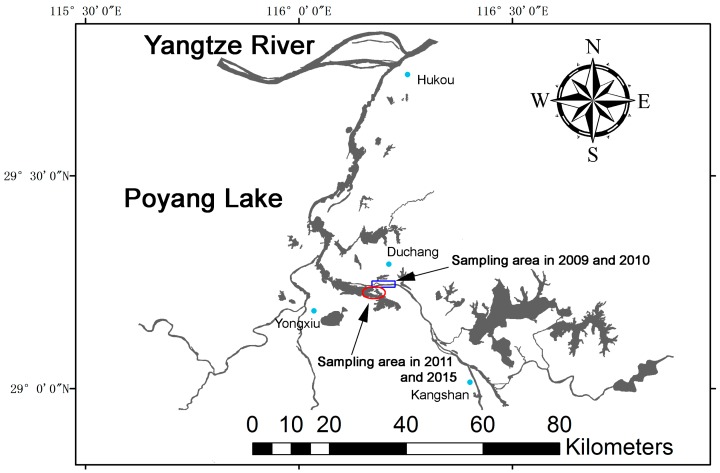
Water coverage of the Poyang Lake in the early spring. Porpoises primarily distribute along the main channel between Hukou and Kangshan, and also in some large sandpit areas between Duchang and Yongxiu. The blue box represents the sampling area in 2009 and 2010. The red circle represents the sampling area in 2011 and 2015.

Groups composed by maternal relationship are very common in marine odontocetes (e.g., long-finned pilot whales (*Globicephala melas*) [30]; belugas (*Delphinapterus leucas*) [31]; sperm whales (*Physeter macrocephalus*) [32]; killer whales (*Orcinus orca*) [33]). For instance, adult female sperm whales associate with sub-adults to form cohesive ‘social units’ that can remain together over several years [32]. Similarly, killer whales are also characterized either by multi-matrilines (resident killer whales [33]) or a single matriline (transient killer whales [5]). In this study, we also detected a maternal line in one YFP group living in the Poyang Lake. Individuals 2011F19, 2011F20, 2011F21, 2011M15, and 2011M16 were found to be grouping together and were subsequently captured in one net. Genetic results showed that this group comprised of four mother-offspring pairs belonging to one maternal linage. The oldest female, 2011F19, is at the top of the maternal linage and mother to two other adult females (2011F20, 2011F21) and a calf (2011M15), and grandmother to calf 2011M16, and the offspring of female 2011F21 (Figure 2). Furthermore, female 2011F20 was confirmed pregnant through B-type ultrasonic inspection. The matrilineal grouping pattern of the YFPs is further supported by the structure of another group (including individuals from 2009M1 to 2009F3) (Table S3). Groups captured in 2009 cannot be treated as natural groups because sound chasing operations during the survey may have disturbed the natural behavior in these porpoises. Nevertheless, our genetic results revealed that adult females 2009F1, 2009F2, and 2009F3 in this group were very closely related, with *r* values close to that of full-sibs (0.4012 to 0.5372). As these three females shared the same mtDNA haplotype (Table S1), we infer that they have high matrilineal relationship. B-type ultrasonic inspection further confirmed these females were all pregnant.

It is interesting that maternally related female YFPs grouping together were all in various reproductive stages. This phenomenon has also been found in other cetacean species, where females with a dependent calf often form nursing groups to reduce unpredictable risk [34]. Indeed, females may form loose associations with related or unrelated females, preferentially associating with other females in similar reproductive states. For example, Möller et al. [35] found that reproductive state seemed to influence associations between female Indo-Pacific bottlenose dolphins (*Tursiops aduncus*), where females with same aged calves within social clusters usually exhibited strong association coefficient.

### 3.3. Dispersal Patterns

Our results revealed that in natural mother-offspring groups, male calves were all <2 years old (0.3–1.7 years old), whereas the female calves ranged from 0.1 to 9.5 years of age. Additionally, three male calves not found in groups with their mothers were all older than two years (6.8-year-old 2009M14, 5.4-year-old 2015M8, and 3.7-year-old 2011M6). Furthermore, two mother-male offspring pairs found trapped in the Yangtze mainstream in January 2014 had also been identified as mothers with their male calves younger than two years old (0.58-year-old and 0.75-year-old, respectively; Ding Wang, unpublished data). This discrepancy could result if male calves disperse from their natal groups at approximately two years old, or at least are not in tight association with their mothers as they may have been at under two years old. Female offspring might associate with their mothers at an older age, and can reproduce or perhaps return to reproduce in the natal group. Still, this association may not be strict as female offspring also emigrate out and raise offspring alone. For example, in this study we found twelve mother-calf pairs that remained alone.

## 4. Materials and Methods

### 4.1. Study Location and Distribution of Porpoises

To investigate the health and social structure of the YFP population living in Poyang Lake, four capture-release surveys were conducted in the spring of 2009–2011, and 2015 (20–24 February 2009; 2–11 March 2010; 21–25 February 2011; and 11–20 March 2015), during the dry season of the lake. During this season, Poyang Lake is reduced to a set of channels together with dispersed sandpit areas (Figure 2). In this season, porpoises mainly distribute along the main channel between Hukou and Kangshan, and also in some large sandpit areas between Duchang and Yongxiu (Figure 2). Since those sandpits are connected to the main channel by shallow waters (depth usually ≤2 m), some porpoises are restricted to those sandpit areas during the entirety of the dry season. In 2009 and 2010, capture-release surveys were conducted in the channel of Duchang County, and in 2011 and 2015, the surveys were conducted in the large sandpit areas located between Duchang and Yongxiu (Figure 2).

### 4.2. Sample Collection

The well-developed “sound chase and net capture” method, which had been specially developed by the Institute of Hydrobiology of the Chinese Academy of Sciences to capture porpoises in the Yangtze main stream or natural reserves, was utilized to capture porpoises in the channel of Poyang Lake during the dry season. Once a group of porpoises (usually ≥2 individuals) was observed by the searching boat (a speed boat equipped with a 40 horsepower engine), chasing boats (consisting of 10–12 fishing boats, each about 12 m long equipped with a 15 horsepower engine) would subsequently arrange themselves in line or curve and move in synchrony keeping a distance of about 50 m to generate underwater noise and form an invisible sound barrier that would force the animals swimming slowly to a shallow water near the shore. Afterward, two net boats (driving either face-to-face or in the opposite direction) would quickly release large-meshed nets to form a large enclosure (about 1 km^2^) to surround the porpoises. The porpoises would then be driven to a smaller area with a radius of approximately 100 m, where small-meshed capture nets would be released quickly to surround the porpoises. All animals were allowed to swim freely in the small enclosure to have enough rest before any further manipulation. After approximately 30 min, the fishermen would draw the capture nets slowly to compress the enclosure until the animals were caught safely. Conversely, because the porpoises were restricted to a relatively small and shallow area (<1 km^2^) in the sandpit areas, the sound chasing operation was rendered unnecessary, leaving only net capture protocols to be used to catch the animals. 

In each capture operation, only a single or a small group of porpoises were captured. After the porpoises were successfully caught and sent to the examination platform, gender was identified, and then each male or female was given a serial ID number (e.g., 2009M1–2009M21, 2009F–2009F8) before proceeding. The serial ID numbers of those porpoises that had been captured in the same net were recorded. If an adult female and a calf had been captured in the same net, a suspected mother-calf pair was then recorded. Reproductive state (pregnancy or lactation) of the adult female was also noted. Body weight and length were measured and recorded. Blood samples were also drawn from the vein in the fluke using a disposable syringe. Blood was anti-coagulated with acid-citrate-dextrose (ACD), and then preserved in liquid nitrogen until DNA extraction. A total of 132 individuals (including 10 recaptured individuals) were captured and sampled: 29 in 2009, 22 in 2010, 46 in 2011 and 35 in 2015. All captured individuals were marked using a unique internal passive intergrated tag label (PIT label; HT850, Hongteng Company, Guangzhou, China), which could be identified via scanning. Group size and individual interaction information were all recorded during capturing (for details see Table S1). 

All capture-release surveys were authorized by the Poyang Lake Fishery and Fishing Administration Office of Jiangxi Province. All sampling was conducted in accordance with the Regulations of the People’s Republic of China for the Implementation of Wild Aquatic Animal Protection (promulgated in 1993), and adhering to all ethical guidelines and legal requirements in China.

### 4.3. DNA Extraction and PCR Amplification

Genomic DNA was isolated using the Whole Genome DNA Extraction Kit (SBS, Shanghai, China) following the manufacturer’s instructions. Twenty-one polymorphic microsatellite loci (simple sequence repeats, *SSR*) were then used in parentage identification. Markers used included *SSR1*, *SSR5*, *SSR8*, *SSR15*, *SSR22*, *SSR40*, *SSR41*, *SSR42*, *SSR51*, *SSR59*, *SSR63*, *SSR69*, *SSR71*, *SSR73*, and *SSR75* from *Neophocaena phocaenoides asiaeorientalis* [36,37,38]; *PPHO130* from *Phocoena phocoena* [39], and *NP391*, *NP404*, *NP409*, *NP464*, and *NP428* from *Neophocaena phocaenoides* [40,41]. PCR was performed in 15-μL reaction volumes containing 1 μL of template DNA, 1.5 μL 10× buffer, 0.7 μM of each primer, 0.25 mM deoxynucleotides (dNTPs), and 0.2 U of *Taq* DNA polymerase (Biostar; Wuhan Tianyuan Huida Biotech Company, China). Amplifications were carried out with conditions consisting of 95 °C for 5 min, followed by 33 cycles of denaturation at 95 °C for 30 s, annealing at 59.5 °C for 30 s and extension at 72 °C for 30 s, with a final extension step at 72 °C for 5 min. PCR products were separated by capillary electrophoresis on an ABI3130XL automated sequencer (Applied Biosystems, Foster City, CA, USA) and alleles were sized against the internal size standard (GeneScan ROX 500, ThermoFisher Scientific, Shanghai, China) using GeneMapperID v3.2 (Applied Biosystems). To minimize scoring error, samples that were homozygous, had low frequency alleles (only appeared in one or two individuals), or exhibited stutter bands, were amplified and genotyped at least three times. We used MICRO-CHECKER version 2.2.3 (Norwich Research Park, Norwick, UK) [42] to check for null alleles, stuttering error, and allele dropout for each locus, at a confidence level of 95%.

A mtDNA control region segment of 597 bp, located at 84–680 bp of the complete control region, was selected due to highly variability [43]. The sequence was amplified with a forward primer (5′-GAA TTC CCC GGT CTT GTA AAC C-3′) and a reverse primer (5′-GGT TTG GGC CTC TTT GAG AT-3′) [44]. PCR amplifications were carried out in 25 μL reactions containing 10–100 ng genomic DNA, 0.6 μM of each primer, 2.5 μL 10× buffer, 0.25 mM dNTPs, and 1 U of *Taq* DNA polymerase (Biostar). Amplifications startedat 95 °C for 5 min, followed by 35 cycles of denaturation at 95 °C for 45 s, annealing at 60 °C for 45 s, and extension at 72 °C for 90 s, with a final extension step at 72 °C for 7 min. PCR products were then purified using a purification kit (PCR Product Purification Kit, BioTeke, Beijing, China) and sequenced in both directions using the PCR primers. Sequencing was performed on an ABI3130 DNA sequencer (Applied Biosystems).

### 4.4. Data Analysis

We assessed measures of genetic diversity of microsatellite loci, including *N*_a_, *H*_o_, *H*_e_, and PIC [45,46], using CERVUS version 3.0 (Field Genetics Ltd., London, UK) [47,48]. The *N*_e-1p_ and *N*_e-2p_ for each microsatellite locus, and the combined values of microsatellite loci for *N*_e-1p_ and *N*_e-2p_, were calculated by using CERVUS version 3.0 [47,48,49]. Hardy-Weinberg equilibrium across all microsatellite loci was assessed via an exact probability test implemented in GENEPOP version 4.0 (Laboratiore de Genetique et Environment, Montpellier, France) [50]. FSTAT version 2.9.3.2 (University of Lausanne, Lausanne, Switzerland) was used to calculate the inbreeding coefficient index *F*_is_ [51]. 

### 4.5. Detecting Potential Parents

We used the age-length formula established by Zhang [52] to calculate the age of each individual. The relationship between age (*x*) and body length (in cm) of male (*L*_m_) and female (*L*_f_) YFP can be calculated as follows:

*L*_m_ = 114.4458 *x*^0.1410^ (♂ ≤ 13.0 years) (1)

and

*L*_f_ = 116.2519 *x*^0.0947^ (♀ ≤ 16.5 years) (2)

Although the age at first reproduction in females remains unknown, Zhang [52] and Wu et al. [53] estimated that YFP females are sexually mature at approximately four years of age and males at 4.5 years of age. In order to not miss potential parents, we considered all female and male porpoises with calculated age ≥3 years old as potential parents. Accordingly, we considered individuals three years younger than the oldest within the population to be potential offspring.

### 4.6. Parentage Analysis

Parentage analysis was conducted using the maximum likelihood (ML) method implemented in program CERVUS version 3.0 [47,48]. This method compares the likelihood of the two most likely mothers or fathers. For each offspring, the difference between the likelihoods of the two most probable mothers or fathers produces a Δ score. Simulations were conducted to estimate the critical values of Δ required to assign parentage with a certain degree of confidence, based on the assumptions made about the population. Parentage assigned at both the 95% and 80% confidence levels were reported, as determined by the critical Δ score. We set parameters for the simulation as follows: the proportion of loci was 1.0, the proportion of potential father and mother were both 20%, the level of potential mistyping was 1%. Simulations were conducted for 100,000 repetitions.

The *r* was also calculated to analyze kinship. The theoretical values between parent-offspring and full-sibs are 0.5; those between half-sibs are 0.25 [21,54]. To calculate *r* estimates, we used the triadic likelihood estimator (TrioML [55]). This estimator computes the relatedness of a dyad in relation to a third reference individual in order to minimize errors stemming from identity-in-state rather than identity-by-descent. It further allows the specification of a genotyping error rate and is bounded between 0 and 1, a more legitimate range than that of other estimators. An evaluation using empirical and simulated data for seven different estimators showed that the TrioML estimator produced the most accurate estimates of kinship [55].

## 5. Conclusions

In this study, group composition and dispersal patterns were studied for the YFP population living in Poyang Lake. To avoid human disturbance to natural groups and guarantee the reliability of the results, we identified natural groups based on both genetic parentage and observational data. Results indicated maternal relationship and reproductive status may be important factors for group composition of females. The dispersal patterns of the YFP showed that male calves may disperse from their mothers at approximately two years old, or at least they were not in tight association with their mothers as they may have been under two years old. Female offspring are observed to stay longer with their mothers and can reproduce in the natal group. 

In the wild, the YFP is difficult to identify and track, and samples used for relatedness studies are difficult to obtain. Therefore, social behavior studies for this population in particular are poor. This study collected information from four capture-release surveys, however, accurate and valuable information is still restricted. For example, calculated parentage was limited, which may be because of the potential high mortality of calves in the Poyang Lake during the dry season, ultimately reducing detectable aspects of social behavior in this study. In addition, although we estimated the dispersal window of male calves, these observations were also limited to few datum points and they should be prudently treated.

## Figures and Tables

**Table 1:** Characteristics of genetic diversity at 21 microsatellite loci for 122 Yangtze finless porpoises in the Poyang Lake.

Locus	*N*_a_	*H*_o_	*H*_e_	*PIC*	*N*_e-1p_	*N*_e-2p_	*F*_is_
*SSR1*	6	0.639	0.629	0.567	0.785	0.629	−0.017
*SSR5*	9	0.795	0.799	0.765	0.580	0.402	0.005
*SSR8*	6	0.800	0.740	0.691	0.678	0.503	−0.082
*SSR15*	11	0.746	0.716	0.660	0.708	0.541	−0.041
*SSR22*	4	0.689	0.665	0.591	0.777	0.628	−0.041
*SSR40*	10	0.746	0.746	0.705	0.657	0.477	0.000
*SSR41*	6	0.425	0.673	0.627	0.736	0.561	0.009
*SSR42*	5	0.610	0.671	0.608	0.751	0.592	0.092
*SSR51*	12	0.780	0.796	0.765	0.574	0.396	0.017
*SSR59*	10	0.854	0.828	0.801	0.522	0.349	−0.031
*SSR63*	7	0.742	0.632	0.559	0.787	0.641	−0.182
*SSR69*	7	0.694	0.611	0.575	0.784	0.606	−0.136
*SSR71*	7	0.529	0.523	0.490	0.848	0.682	−0.013
*SSR73*	6	0.782	0.793	0.756	0.599	0.420	0.013
*SSR75*	16	0.672	0.644	0.625	0.733	0.54	−0.044
*NP391*	12	0.415	0.475	0.441	0.877	0.723	0.128
*NP404*	5	0.504	0.555	0.459	0.844	0.736	0.077
*NP409*	9	0.664	0.748	0.706	0.652	0.476	0.113
*NP428*	5	0.374	0.361	0.345	0.930	0.792	−0.035
*NP464*	9	0.628	0.764	0.724	0.634	0.456	0.178
*PPHO130*	9	0.795	0.787	0.752	0.596	0.419	−0.010
Mean of 21 loci	8.1	0.661	0.674	0.629	Combined 7.20 × 10^−4^	Combined 2.17 × 10^−6^	0.0002 (*p* > 0.05)

*N*_a_: Number of alleles per locus, *H*_o_: observed heterozygosity, *H*_e_: expected heterozygosity, PIC: polymorphic information content, *N*_e-1p_: average non-exclusion probability when no parents were known, *N*_e-2p_: or when one parent was known, *F*_is_: inbreeding coefficient index.

## Supplementary Materials

Supplementary materials can be found at http://www.mdpi.com/1422-0067/17/8/1268/s1.
