# Supplementary material for: Parentage-Based Group Composition and Dispersal Pattern Studies of the Yangtze Finless Porpoise Population in Poyang Lake
Source: Int J Mol Sci. 2016 Aug 11;17(8):1268. doi: 10.3390/ijms17081268 (PMC5000666; doi:10.3390/ijms17081268)
Supplement: Supplementary file 1 [file ijms-17-01268-s001.pdf]

Supplementary Materials: Parentage-Based Group Composition and Dispersal Pattern Studies of the Yangtze Finless Porpoise Population in Poyang Lake

Minmin Chen, Yang Zheng, Yujiang Hao, Zhigang Mei, Kexiong Wang, Qingzhong Zhao, Jinsong Zheng and Ding Wang

Table S1. Mitochondrial DNA (mtDNA) haplotypes and their distribution in porpoises in Poyang Lake.

Haplotype	Individuals
NAACR-Hap1	2009F4, 2009M5, 2009M7, 2009M8, 2009M12, 2009M14, 2009M16, 2009M20, 2009F6, 2009F7, 2009F8, 2010M2, 2010F5, 2010F7, 2010F8, 2010M5, 2010M6, 2010M8, 2011M1, 2011F4, 2011M3, 2011M7, 2011M8, 2011F7, 2011F9, 2011F14, 2011F15, 2011M13, 2011M19, 2011F24, 2015F1, 2015F2, 2015F4, 2015F5, 2015M1, 2015F6, 2015F7, 2015F8, 2015F9, 2015M3, 2015M4, 2015F13, 2015F14, 2015F15, 2015M6, 2015M7, 2015M8, 2015F17, 2015M10, 2015M11, 2015F19, 2015F20, 2015F22
NAACR-Hap2	2009M1-2009M4, 2009F1-2009F3, 2009M6, 2009M9, 2009M10, 2009M13, 2009M15, 2009M17, 2009M18, 2009M19, 2009M21, 2010F1, 2010F2, 2010F3, 2010M3, 2010M4, 2010F9, 2010F11, 2010F12, 2010F13, 2011F1, 2011F2, 2011M2, 2011F3, 2011F5, 2011M4, 2011M6, 2011F6, 2011M9, 2011M10, 2011F10-2011F13, 2011M12, 2011F16-2011F22, 2011M15-2011M17, 2011M18, 2011F25, 2011M20, 2011M21, 2015M02, 2015F10, 2015F11, 2015F12, 2015M5, 2015M9, 2015F18, 2015M12, 2015F21, 2015M13, 2015M14
NAACR-Hap8	2009M4, 2009M11, 2010M1, 2011F8, 2011M11

Table S2. Twenty-one mother-offspring pairs and six father-offspring pairs detected by CERVUS from the Yangtze finless porpoise population living in Poyang Lake.

Parent	Sex/Age (Years)	mtDNA Haplotypes	Offspring	Sex/Age (Years)	mtDNA Haplotypes	Relatedness Index *r*	Confidence Level	Relation from Field Data
2015F2	F/5.2	NAACR-Hap1	2015F1	F/0.2	NAACR-Hap1	0.4825	95%	Suspected
2015F18	F/12.8	NAACR-Hap2	2015F10	F/1.8	NAACR-Hap2	0.5480	95%	Unknown
2015F11	F/10.3	NAACR-Hap2	2015F12	F/2.5	NAACR-Hap2	0.4077	95%	Suspected
2015F19	F/4.5	NAACR-Hap1	2015F20	F/0.4	NAACR-Hap1	0.4932	95%	Suspected
2011F2	F/9.5	NAACR-Hap2	2011F1	F/2.5	NAACR-Hap2	0.5408	95%	Suspected
2011F4	F/11.9	NAACR-Hap1	2011M3	M/1.7	NAACR-Hap1	0.4848	95%	Suspected
2011F4	F/11.9	NAACR-Hap1	2015M8	M/5.4	NAACR-Hap1	0.2924	95%	Unknown
2011F12	F/5.2	NAACR-Hap2	2011F10	F/0.6	NAACR-Hap2	0.4349	95%	Suspected
2011F19	F/15.8	NAACR-Hap2	2011M15	M/1.7	NAACR-Hap2	0.3809	95%	Suspected
2011F19	F/15.8	NAACR-Hap2	2011F20	F/6.1	NAACR-Hap2	0.4892	95%	Unknown
2011F19	F/15.8	NAACR-Hap2	2011F21	F/9.5	NAACR-Hap2	0.5000	95%	Unknown
2011F21	F/9.5	NAACR-Hap2	2011M16	M/1.1	NAACR-Hap2	0.4806	95%	Suspected
2011F23	F/8.0	NAACR-Hap2	2011F22	F/0.8	NAACR-Hap2	0.3531	95%	Suspected
2011F24	F7.6	NAACR-Hap1	2011M19	M/0.6	NAACR-Hap1	0.3712	95%	Suspected
2011F25	F/6.6	NAACR-Hap2	2011M20	M/0.3	NAACR-Hap2	0.4057	95%	Suspected
2010F6	F/11.1	NAACR-Hap2	2011M6	M/3.7	NAACR-Hap2	0.5000	95%	Unknown
2010F5	F/13.7	NAACR-Hap1	2009M14	M/6.8	NAACR-Hap1	0.3749	95%	Unknown
2010F11	F/7.1	NAACR-Hap2	2010F12	F/0.1	NAACR-Hap2	0.5551	95%	Suspected

Table S2. *Cont.*

Parent	Sex/Age (Years)	mtDNA Haplotypes	Offspring	Sex/Age (Years)	mtDNA Haplotypes	Relatedness Index *r*	Confidence Level	Relation from Field Data
2009F7	F/≥16.5	NAACR-Hap1	2009F8	F/1.2	NAACR-Hap1	0.5000	95%	Suspected
2009F7	F/≥16.5	NAACR-Hap1	2015F2	F/5.2	NAACR-Hap1	0.3115	95%	Unknown
2009F7	F/≥16.5	NAACR-Hap1	2015M7	M/0.9	NAACR-Hap1	0.5000	95%	Suspected
2015M3	M/7.8	/	2015F12	F/2.5	/	0.4333	95%	Unknown
2009M5	M/≥13.0	/	2011F24	F/7.6	/	0.2795	80%	Unknown
2009M5	M/≥13.0	/	2009M10	M/9.8	/	0.2295	80%	Unknown
2009M5	M/≥13.0	/	2009M11	M/3.7	/	0.3805	80%	Unknown
2009M5	M/≥13.0	/	2009F6	F/3.0	/	0.4923	95%	Unknown
2009M2	M/8.2	/	2009F8	F/1.2	/	0.5032	95%	Unknown

Table S3. Information of samples and the relationship of individuals captured in one net.

Code	Sex	Body Length (cm)	Age (Years)	Capture	Relatedness Index *r*	Parentage
				**2009**		
2009M1	M	104	0.5	One net	2009F1-F2, *r* = 0.5372<br>2009F1-F3, *r* = 0.4012<br>2009F2-F3, *r* = 0.4116	/
2009F1	F	140	7.1			
2009F2	F	145	10.3			
2009M2	M	154	8.2			
2009M3	M	129.5	2.4			
2009M4	M	154	8.2			
2009F3	F	144	9.5			
2009F4	F	142	8.2	One net	/	/
2009F5 (2009F1)	F	140	7.1			
2009M5	M	170	≥13			
2009M6	M	127.5	2.1			
2009M7	M	150	6.8			
2009M8	M	127	2.0	One net	2009M10-M11, *r* = 0.2102	/
2009M9	M	136	3.4			
2009M10	M	158	9.8			
2009M11	M	138	3.7			
2009M12	M	122	1.5	One net	/	/
2009M13	M	154	8.2			
2009M14	M	150	6.8	One net	2009M14-M16, *r* = 0.2681	/
2009M15	M	153	7.8			
2009M16	M	158	9.8			
2009M17	M	127	2.0	One net	/	/
2009M18	M	157	9.4			
2009M19	M	128	2.2	One net	2009M20-M21, *r* = 0.2078	/
2009M20	M	125	1.8			
2009M21	M	146	5.6			
2009F6	F	129	3.0		/	/

Table S3. *Cont.*

Code	Sex	Body Length (cm)	Age (Years)	Capture	Relatedness Index *r*	Parentage
2009F7	F	152.5	≥16.5	One net	*r* = 0.5000	Mother-calf
2009F8	F	119	1.2			
			**2010**			
2010F1	F	138	6.1		/	/
2010F2	F	95	0.1		/	/
2010M1	M	149	6.5		/	/
2010M2	M	104	0.5	One net	2010F2-M2, *r* = 0.3368	/
2010F3	F	100	0.2			
2010F4 (2010F2)	F	95	0.1			
2010F5	F	149	13.7		/	/
2010M3	M	127	2.0	One net	/	/
2010M4	M	114	0.9			
2010F6 (2010F1)	F	138	6.1			
2010F7	F	128	2.7			
2010F8	F	146	11.0			
2010M5	M	151.5	7.3		/	/
2010F9	F	119	1.2		/	/
2010M6	M	127	2.1	One net	/	/
2010F10 (2010F9)	F	119	1.2			
2010M7 (2009M20)	M	128	2.2			
2010F11	F	140	7.1	One net	*r* = 0.5551	Mother-calf
2010F12	F	98	0.1			
2010F13	F	141	7.6	One net	/	/
2010F14 (2010F9)	F	119	1.2			
2010M8	M	119	1.3		/	/

Table S3. *Cont.*

Code	Sex	Body Length (cm)	Age (Years)	Capture	Relatedness Index *r*	Parentage
**2011**						
2011M1	M	168	≥13		/	/
2011F1	F	127	2.5	One net	*r* = 0.5408	Mother-calf
2011F2	F	144	9.5			
2011M2	M	166	≥13	One net	/	/
2011F3	F	138	6.1			
2011F4	F	147	11.9	One net	*r* = 0.4848	Mother-calf
2011M3	M	124	1.7			
2011F5	F	124	1.9	One net	2011F5-M4, *r* = 0.2273	/
2011M4	M	124	1.7			
2011M5 (2009M11)	M	140	4.1			
2011M6	M	137	3.5			
2011M7	M	113	0.9			
2011F6	F	140	7.1			
2011M8	M	113	0.9			
2011F7	F	118	1.1	One net	/	/
2011M9	M	124	1.7			
2011F8	F	130	3.2	One net	2011F8-M11, *r* = 0.4000	/
2011F9	F	128	2.7			
2011M10	M	149	6.5			
2011M11	M	116	1.1			
2011F10	F	112	0.6	One net	2011F13-F11, *r*= 0.4349	2011F13-F11 Mother-calf
2011F11	F	123	1.8			
2011F12	F	136	5.2			
2011F13	F	139	6.6			
2011F14	F	140	7.1	One net	/	/
2011F15	F	129	3.0			

Table S3. *Cont.*

Code	Sex	Body Length (cm)	Age (Years)	Capture	Relatedness Index *r*	Parentage
2011M12	M	108	0.6	One net	2011F19-M15, *r* = 0.3809<br>2011F19-F20, *r* = 0.4892<br>2011F19-F21, *r* = 0.5000<br>2011F21-M16, *r* = 0.4806<br>2011M12-F16, *r* = 0.2578<br>2011F17-F20, *r* = 0.2086<br>2011F17-M15, *r* = 0.2743	2011F19-M15 Mother-calf;<br>2011F19-F20 Mother-calf;<br>2011F19-F21 Mother-calf;<br>2011F21-M16 Mother-calf
2011M13	M	146	5.6			
2011M14 (2011M8)	M	113	0.9			
2011F16	F	137	5.6			
2011F17	F	148	12.8			
2011F18	F	119	1.2			
2011F19	F	151	15.8			
2011M15	M	124	1.7			
2011M16	M	116	1.1			
2011F20	F	138	6.1			
2011F21	F	144	9.5			
2011F22	F	115	0.8	One net	*r* = 0.3531	Mother-calf pair
2011F23 (2009F1)	F	141.5	8.0			
2011M17	M	148	6.1	One net	2011F24-M19, *r* = 0.3712<br>2011F25-M20, *r* = 0.4057	Two mother-calf
2011M18	M	150	6.8			
2011M19	M	107	0.6			
2011F24	F	141	7.6			
2011F25	F	139	6.6			
2011M20	M	100	0.3			
2011M21	M	152	7.4			
**2015**						
2015F1	F	101	0.2	One net	*r* = 0.4825	Mother-calf
2015F2	F	136	5.2			
2015F4	F	115	0.9	One net	*r* = 0.5000	Mother-calf
2015F5	F	147	11.9			
2015M1	M	122	1.6	One net	/	
2015F6	F	104/108	0.5			
2015F7	F	131	3.5			

Table S3. *Cont.*

Code	Sex	Body Length (cm)	Age (Years)	Capture	Relatedness Index *r*	Parentage
2015M2	M	108	0.7	One net	/	
2015F8	F	149	13.7			
2015F9	F	103	0.3			
2015M3	M	153	7.8	One net	2015M3-M4, r = 0.2338<br>2015F10-M4, r = 0.3206	
2015F10	F	123	1.8			
2015M4	M	161/158	9.8			
2015F11	F	154/145	10.3	One net	r = 0.4077	Mother-calf
2015F12	F	127	2.5			
2015F13	F	147	11.9	One net	2015F13-F14, r = 0.3807	
2015F14	F	105	0.3			
2015M5	M	124	1.8			
2015F15	F	134	4.5			
2015M6	M	144	5.1	One net	2015F16-M7, r = 0.5000	Mother-calf
2015F16 (2009F7)	F	158	≥16.5			
2015M7	M	112	0.9			
2015PYM08	M	145	5.4			
2015F17	F	125	2.2	One net	/	
2015M9	M	160	10.8			
2015M10	M	152	7.5			
2015PYF18	F	148	12.8			
2015M11	M	135/129	2.3			
2015M12	M	138	3.8			
2015F19	F	134	4.5	One net	r = 0.4932	Mother-calf
2015F20	F	107	0.4			
2015F21	F	131	3.5	One net	2015F21-M14, r = 0.2945<br>2015M13-M14, r = 0.3568	
2015M13	M	154	8.2			
2015M14	M	129	2.3			
2015F22	F	138	6.1			

Individuals with two codes were recaptured. The code in brackets was given when the animal was captured for the first time.
